# An Interesting Case of Paroxysmal Nocturnal Hemoglobinuria With Renal Involvement

**DOI:** 10.7759/cureus.63917

**Published:** 2024-07-05

**Authors:** Shawn Keating, Riddhi Machchhar, Ujjwala Jain, Gabriela Naronowicz, Jordan Lipschutz

**Affiliations:** 1 Internal Medicine, Ocean University Medical Center, Brick, USA

**Keywords:** treatment, biopsy, paroxysmal nocturnal hemoglobinuria, dialysis, hematuria

## Abstract

Paroxysmal nocturnal hemoglobinuria (PNH) is an uncommon genetic disorder that affects red blood cell production, causing symptoms like fatigue, abdominal pain, and shortness of breath. This condition can also result in dark urine and an increased risk of infections. Diagnosis of PNH involves genetic testing and flow cytometry, which can confirm the presence of the condition. Once a diagnosis is confirmed, personalized treatment plans should be developed to effectively manage the symptoms and improve the patient's quality of life. Treatment options for PNH may include bone marrow transplantation, blood transfusions, and the use of recombinant monoclonal antibody, eculizumab. Regular monitoring is also essential to identify and manage any complications that may arise due to this condition. With proper management and treatment, patients with PNH can lead a healthy and fulfilling life. In this case study, we present a young adult male with PNH who also suffers from renal failure, highlighting the importance of personalized care and ongoing monitoring for this complex condition.

## Introduction

Paroxysmal nocturnal hemoglobinuria (PNH) is a unique hematological condition that destroys red blood cells, resulting in hemolysis, thrombosis, and impaired bone marrow function. It is caused by a mutation in the PIG-A gene, which leads to a deficiency of specific proteins on the cell membrane, including glycosylphosphatidylinositol-anchored proteins (GPI-APs), CD55, and CD595 [1]. This deficiency makes affected cells vulnerable to complement-mediated lysis, leading to the diverse clinical manifestations observed in PNH. PNH can range from mild fatigue and dark-colored urine to life-threatening thrombotic events and bone marrow failure [2]. This case report aims to comprehensively analyze a recent clinical encounter with a patient diagnosed with PNH, highlighting the diagnostic challenges, treatment strategies, and outcomes observed. Through this case, we aim to enhance our understanding of this complex disorder, facilitate methods for early recognition, and promote optimal management of PNH, including the current standard of care, eculizumab.

## Case presentation

A 28-year-old male patient with a medical history of recurrent kidney stones, hematuria, and erectile dysfunction presented to the emergency department (ED) with jaundice and complaints of nausea, vomiting, and lower abdominal discomfort for one day. Notably, the patient reported that his urine had been progressively dark for the past seven months and denied any associated symptoms of fever or chills. Of significance, the patient had been previously hospitalized a year and a half before with COVID-19, acute renal failure, anemia, and thrombocytopenia, receiving care from hematology and nephrology teams. Subsequently, the patient has been regularly followed up by nephrology. During the previous hospitalization, serologies were negative for inciting factors of acute renal failure, while a kidney biopsy revealed tubular degenerative changes with severe prominent pigmented casts, raising concern for hemoglobinuric acute tubular necrosis (ATN). A bone marrow biopsy performed during hospitalization was also negative for immune complex disease and nonindicative of a definite diagnosis. As a result, further evaluation was recommended, and the patient received a referral to a facility with novel therapies for renal disease.

After the patient's hospitalization, they began to experience recurring episodes of hematuria, prompting visits to the emergency department. Despite the presence of gross hematuria in the samples obtained, clotting was not observed. These episodes were attributed to various causes, including chronic kidney stones, urinary tract infections, or viral illnesses. It is worth noting that the patient typically denied experiencing dysuria, abdominal pain, or flank pain, and their symptoms - which were mostly confined to hematuria - were usually resolved with a brief course of antibiotics.

Upon current presentation to the ED, his vitals were as follows: blood pressure of 117/65 mmHg, heart rate of 60 bpm, temperature of 98°F, respiratory rate of 18, and spO2 at 98%. His labs revealed anemia, increased creatinine (2.73 mg/dL), blood urea nitrogen (BUN) (32), and total bilirubin (6.9 mg/dL) (Table 1).

**Table 1:** Lab values on admission.

Complete blood count (CBC)	Reference	Values
White blood cells	4.5 - 11.1 (x10^3^/uL)	5.7
Red blood cells	4.50 - 5.30 (x10^6^/uL)	3.33
Hemoglobin	13.2 - 17.5 g/dL	8.6
Hematocrit	40% - 53%	28.4
Mean corpuscular volume	80 - 100 fL	85.3
Red cell distribution width	11.5 - 14.5%	18.3
Platelet count	140 - 450 (x10^3^/uL)	193
Neutrophils	1.8 - 8.0 (x10^3^/uL)	82.7
Lymphocytes	1.5 - 3.5 (x10^3^/uL)	9.4
Monocytes	0.0 - 1.0 (x10^3^/uL)	7.2
Complete metabolic panel (CMP)		
Blood urea nitrogen (BUN)	5 - 25 mg/dL	32
Creatinine	0.61 - 1.24 mg/dL	2.73
Glomerular filtration rate (GFR)	>60 mL/min/1.73 m^2^	28
Alkaline phosphatase	38 - 126 U/L	89
Albumin	3.5 - 5.0 g/dL	4.2
Total bilirubin	<1.3 mg/dL	6.9
Direct bilirubin	<0.2 mg/dL	1.0
Indirect bilirubin	<1.1 mg/dL	5.9
Aspartate aminotransferase (AST)	10 - 42 U/L	380
Alanine aminotransferase (ALT)	10 - 60 U/L	70

His alpha-1 antitrypsin was mildly elevated to 205 with serum C3 and C4 negative. His urine sample reflected turbid brown urine with some red blood cells (RBCs), amorphous crystals, and granular casts. He was provided ondansetron and intravenous fluids (IVF) in the ER. The patient was seen by nephrology and was perceived to be jaundiced due to possible hemolytic anemia, given the indirect hyperbilirubinemia and history of elevated lactate dehydrogenase (LDH) with levels in the 1000s. A thorough workup was pursued with autoimmune and subsequent viral hepatitis markers obtained.

The negative Coombs test and worsening renal failure in the face of recurrent acute kidney injury (AKI) increased suspicion of PNH and the likelihood of advancing hemoglobinuric ATN (Table 2).

**Table 2:** Lab values after admission.

	Reference	Day 8	Day 7	Day 6	Day 5	Day 4	Day 3	Day 2	Day 1
Complete blood count (CBC)									
White blood cells	4.5 - 11.1 (x10^3^/uL)	7.5	6.0	5.8	6.2	5.9	5.0	4.1	4.2
Red blood cells	4.50 - 5.30 (x10^6^/uL)	2.85	2.62	2.70	2.63	2.71	2.51	2.88	2.42
Hemoglobin	13.2 - 17.5 g/dL	7.9	7.2	7.4	7.4	7.6	6.9	7.7	6.5
Hematocrit	40% - 53%	24.7	23.0	23.7	22.7	23.3	21.9	25.2	20.7
Mean corpuscular volume	80 - 100 fL	86.7	87.8	87.8	86.3	86.0	87.3	87.5	87.5
Red cell distribution width	11.5 - 14.5%	18.0	18.4	18.3	18.6	18.6	18.8	18.4	18.7
Platelet count	140 - 450 (x10^3^/uL)	81	84	61	73	109	161	152	156
Neutrophils	1.8 - 8.0 (x10^3^/uL)	5.0	4.0	4.0	4.7	4.1	3.7	2.5	3.0
Lymphocytes	1.5 - 3.5 (x10^3^/uL)	1.1	1.0	0.9	0.6	0.9	0.6	1.0	0.8
Monocytes	0.0 - 1.0 (x10^3^/uL)	1.1	0.8	0.7	0.9	0.8	0.6	0.6	0.5
Complete metabolic panel (CMP)									
Blood urea nitrogen (BUN)	5 - 25 mg/dL	21	35	30	36	50	73	66	55
Creatinine	0.61 - 1.24 mg/dL	4.46	5.63	4.72	5.50	6.69	7.81	6.90	5.20
Glomerular filtration rate (GFR)	>60 mL/min/1.73 m^2^	16	12	15	12	10	8	10	13
Alkaline phosphatase	38 - 126 U/L	80	72	73	74	79	76	78	64
Albumin	3.5 - 5.0 g/dL	2.9	2.8	2.9	3.1	3.2	3.3	3.6	3.2
Total bilirubin	<1.3 mg/dL	1.2	1.3	1.2	1.1	1.8	2.0	3.1	4.7
Direct bilirubin	<0.2 mg/dL			0.3					
Indirect bilirubin	<1.1 mg/dL								
Aspartate aminotransferase (AST)	10 - 42 U/L	24	23	20	21	33	81	231	305
Alanine aminotransferase (ALT)	10 - 60 U/L	22	21	23	29	36	46	62	59
Total creatinine kinase	22 - 232 (iU)/L								477
Lactate dehydrogenase	91 - 200 U/L			945	1,179				

**Figure 1:**
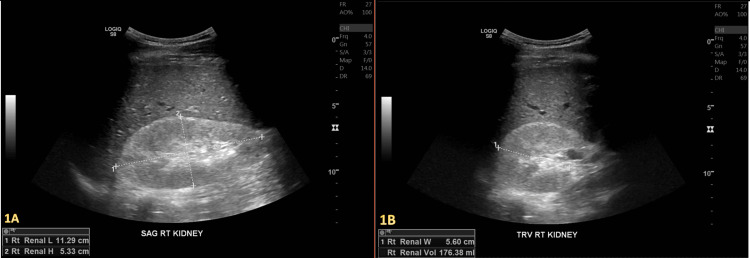
Sagittal (A) and transverse (B) views show a partially visualized right kidney with increased right renal cortical echogenicity, suggestive of intrinsic medical renal disease. No hydronephrosis in the right kidney was noted.

**Figure 2:**
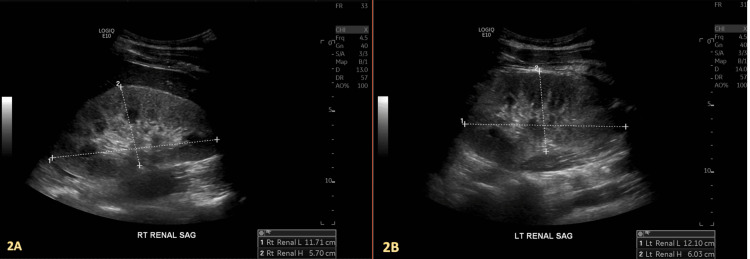
The right renal sagittal view (A) and left renal sagittal view (B) show increased renal cortical echogenicity with poor corticomedullary differentiation, suggestive of intrinsic medical renal disease. No evidence of hydronephrosis in either kidney was noted.

An intrinsic medical renal disease was discovered on a right upper quadrant ultrasound (US) (Figure 1), followed by a bilateral renal ultrasound, which revealed increased echogenicity consistent with chronic kidney disease (Figure 2).

The patient's case was thoroughly discussed with the nephrologist overseeing his outpatient care and the specialty renal clinic, and it was decided that immediate treatment with eculizumab was necessary. The patient was also scheduled for hemodialysis through a temporary permcath, provided with one unit of packed red blood cells (PBRCs) due to a declining hemoglobin count, and given the meningitis vaccine and penicillin prophylaxis as a precautionary measure. Another bone marrow biopsy was also ordered. The patient was monitored for clinical improvement after dialysis, and discharge plans were initiated accordingly. Toward the end of the hospitalization, the presence of a positive PNH clone was identified in the neutrophils (72.49%), monocytes (74.76%), and RBCs (total: 15.36%, type II: 2.59%, type III: 12.77%), and autoimmune markers such as anti-smooth muscle antibody (ASMA) and antimitochondrial antibodies (AMA) were negative. Of significance, flow cytometry results, excluding CD55 and CD59 expression, confirmed the diagnosis of PNH in the relatively appropriate clinical setting (Tables 3, 4).

**Table 3:** Flow cytometry - paroxysmal nocturnal hematuria (PNH) assay. Methodology: Antibodies used were directed against CD45, glycophorin A, CD59, CD24, CD14, CD15, and CD64, as well as fluorescein-labeled proaerolysin (FLAER). The above-estimated percentages are based on the scattergram of CD45, forward and/or side scatter, or phenotypic cluster analysis. This high-sensitivity assay can detect as few as one in 10,000 glycosylphosphatidylinositol (GPI)-deficient cells in a mixed population and can be used for sequential monitoring of disease levels in patients with paroxysmal nocturnal hemoglobinuria (PNH). Reference: Borowitz MJ, Craig FE, Digiuseppe JA, et al.: Guidelines for the diagnosis and monitoring of paroxysmal nocturnal hemoglobinuria and related disorders by flow cytometry. Cytometry B Clin Cytom. 2010, 78:211-30. DOI: 10.1002/cyto.b.20525.

Interpretation	A PNH clone is identified within the neutrophils (72.49%), monocytes (74.76%), and RBC (total: 15.36%, type II: 2.59%, type III: 12.77%). These findings are consistent with a diagnosis of PNH. Any potential difference in clone size between the WBCs and the RBCs may be due to hemolysis and/or recent transfusion. (Reference: www.pnhsource.com/physicians)
Specimen type	Peripheral blood
Viability (%)	89%
Populations gated	Erythrocytes/granulocytes/monocytes
Abnormal cells detected	Yes
% Abnormal	Type I: Normal CD59 level - 84.71%. Type II: Partial CD59 deficiency - 2.59%. Type III: Complete CD59 deficiency - 12.77%
Granulocytes	FLAER/CD24 deficiency - 72.49%
Monocytes	FLAER/CD14 deficiency - 74.76%
Number of markers	8

**Table 4:** Reticulocyte count.

	Reference	Day 6	Day 2	Hospitalization 1.5 years ago
Reticulocyte count	0.40% - 2.50%	5.35	4.31	1.89
Immature reticulocyte fraction	2.3% - 17.5%	22.0	32.1	11.2
Absolute reticulocytes	0.010 - 0.110 (x10^6^/uL)	0.145	0.122	0.056
Reticulocyte hemoglobin	82.2 - 36.6 pg	30.1	30.1	31.9

The patient responded well to hemodialysis treatments along with eculizumab and was discharged home once deemed medically stable, with scheduled appointments for outpatient dialysis and nephrology follow-up.

## Discussion

This case report examines a patient who experienced PNH and later suffered from kidney failure requiring dialysis. Research has revealed that PNH and other conditions that cause hemolytic anemia, for example, sickle cell, can lead to varying degrees of kidney damage [3,4]. The accumulation of heme, a toxic byproduct of hemoglobin breakdown, can cause inflammation and damage to the renal tubules, leading to a decline in kidney function over time [3]. In this case, the patient's kidney failure was likely facilitated by an assumption that the changes in their urine were caused by a separate medical condition and not seeking medical attention promptly. PNH is a rare disease affecting a small percentage of the population, with eight and 38 people per million impacted [4]. According to the Centers for Disease Control and Prevention (CDC), PNH impacts around 1-1.5 people per million individuals globally, although certain regions have higher incidence rates. The disease can cause severe health complications, including anemia, thrombophilia, and bone marrow failure (BMF) [4-6]. PNH is classified as a hematologic disorder and arises from acquired somatic mutations within hematopoietic progenitor cells. These mutations lead to the absence of cellular complement regulators CD55 and CD59 due to defective glycosylphosphatidylinositol (GPI) anchors. GPI-deficient cells in PNH patients are susceptible to constant lysis by the terminal complement complex (TCC), called membrane attack complex (MAC; C5b-9). This susceptibility is due to the partial or total absence of CD59, which accelerates the decay of C3 convertases [1,4-6].

In Asia, countries such as Japan, Korea, and China have a higher prevalence of PNH compared to Western countries like the United States, Spain, and the United Kingdom. The International PNH Registry, established in 2003, collects comprehensive data on the natural history of PNH and provides valuable epidemiology data. As of 30 June 2012, the registry included 1,610 patients from 273 centers in 25 countries, with 92.5% of patients from Europe and North America and 87.5% of patients of white ethnicity [5]. The age range most prominently represented was 30-59 years, with 54.6% of the registry population falling within this range. PNH is rare in children and tends to manifest in the teenage years. There is a slight female predominance, with 54.4% of patients being female overall [5]. However, in some countries in Asia, men have more access to medical care than women, resulting in a lower proportion of women diagnosed with PNH. Despite limitations to the data from the International PNH Registry, it provides crucial epidemiological insights into PNH and its prevalence rates. Additionally, studies have shown that PNH-associated thrombotic events occur more frequently in Western countries than in Asian countries, with rates up to 30% and <15%, respectively [4-6]. While the epidemiology of PNH-associated BMF is not as well described, it is hypothesized that the Asian population may suffer from this condition due to the increased prevalence of aplastic anemia [5].

As noted, the International PNH Registry is a crucial source of clinical information regarding the symptoms and disease burden experienced by patients suffering from PNH. To assess the impact of this condition on various aspects of patient lives, the registry collects patient-reported symptoms at enrollment through assessments, including those related to quality of life and employment status [4-6]. A review of the baseline data from the registry indicates that out of 856 registered patients, 93.3% reported at least one symptom associated with PNH [5]. PNH patients experience a range of clinical symptoms due to intravascular hemolysis (IVH) and complement-dependent thrombophilia [4-6]. While complement inhibitors can help address some symptoms, others, such as thrombocytopenia, neutropenia, and anemia resulting from underlying BMF, remain unresolved [4]. Untreated PNH patients commonly reported symptoms of fatigue (80%), dyspnea (64%), headache (63%), and hemoglobinuria (62%). Additionally, 38% of male patients experienced erectile dysfunction [4,5]. In 91.4% of cases, patients reported at least one additional symptom besides fatigue, which was the sole complaint in only 2% of patients [5]. These symptoms may be associated with thrombosis and/or ischemia and can worsen with infections, exercise, surgery, vaccination, and other complement-stimulating situations [4]. Additionally, patients may experience phases of jaundice or overt hemoglobinuria [4]. Many of these symptoms of untreated PNH were exhibited in our patient at multiple ER visits. If left undiagnosed for too long, PNH can also lead to secondary complications such as kidney failure [3,4], as seen in our patient, and pulmonary hypertension, or Budd-Chiari syndrome [4].

While clinical evaluation helps diagnose PNH, laboratory testing, specifically flow cytometry analysis of CD55 and CD59 expression, enhances diagnostic accuracy and provides valuable information for treatment decisions. In suspected cases of PNH, flow cytometry testing should be considered to confirm the diagnosis and assess the disease's severity. This approach helps ensure appropriate treatment and that patients receive the care they need to manage their condition effectively. The deficiency of complement regulatory proteins is confirmed through this technique, providing quantitative data on CD55 and CD59 expression levels that can aid in disease monitoring and risk stratification [7,8]. However, it is worth noting that the absence of CD55 and CD59 testing in the flow cytometry panel can result in a missed opportunity to establish a definitive diagnosis and assess the severity of the PNH clone. In our case, where CD55 and CD59 testing is unavailable, the diagnosis depended solely on clinical evaluation, including typical symptoms like hemoglobinuria and anemia, and excluding bone marrow disease through biopsy [7].

In the 1990s, individuals diagnosed with PNH had a median survival rate of approximately 10 years. However, the advent of eculizumab therapy in 2002 and clinical use in 2007 led to a significant increase in this survival rate, extending it to over 20 years [4-6]. This treatment to reduce IVH, decrease LDH levels, prevent recurrent blood transfusion intervention, and mitigate the risk of thrombosis has revolutionized the management of PNH, allowing patients to lead relatively normal lives [4-6]. However, it is crucial to note that patients with hemolytic PNH who receive eculizumab have a better outlook than those with a more severe BMF component, such as aplastic anemia, as eculizumab does not address the underlying production deficit in the bone marrow [5]. In the International PNH Registry, out of the 122 patients who have passed away since enrolling, approximately 11.7% of these deaths were due to BMF in patients who met the diagnostic criteria for both PNH and aplastic anemia [5]. According to data from the registry, it is estimated that roughly 75% of PNH patients receive eculizumab therapy [5] with pegcetacoplan introduced in 2021 as the first proximal complement inhibitor (C3-inhibition/ pegylated cyclic tridecapeptide) [4,6]. Complement component C3 is a crucial factor in controlling the activation and amplification of complement. There are therapeutic options available for inhibiting C3 activation, such as compstatin and its PEGylated derivative pegcetacoplan, which prevent downstream complement reactions and avoid amplification via the complement alternative pathway (CAP) [4,6].

Development of proximal complement therapeutics occurred because of the need to control C3-mediated extravascular hemolysis (EVH), exposed by C5 inhibition, which increasingly poses a significant risk for patients with PNH [6]. Initially, there was concern that interfering with the upstream pathway could increase the risk of opportunistic infections [4,6]. However, infectious complications are negligible with the *Neisseria meningitidis* vaccine, extended coverage vaccinations, *Haemophilus influenzae* and streptococcal pneumonia, and concurrent prophylactic antibiotic usage [4,6]. All proximal complement inhibitors currently require extended vaccination schedules to mitigate the risk of infections [6], this precaution is visible in the treatment plan of our young male patient.

The future for managing and treating PNH is continuously evolving and improving. Given that eculizumab has frequent dosing or requires increased dosages with the continued risk of breakthrough hemolysis (BTH), there have been progressive strides to evaluate further cost-effective, more accessible, and easily accessible treatments. Multiple C5 inhibitors were assessed in the PNH field, but some did not complete phase 3 trials or were terminated for undisclosed reasons. Tesidolumab, a humanized IgG1/λ anti-C5 monoclonal antibody, was tested on PNH patients with and without a C5 polymorphism, demonstrating effectiveness and safety [6], but no further data are available. C5 inhibitors with long-acting properties, like ravulizumab and crovalimab, can be more convenient because they require less frequent administration due to their long half-life and low-volume subcutaneous injections [4,6]. Ravulizumab, for example, is given every eight weeks during the maintenance phase and is being used as a replacement for eculizumab in several countries. While the risk of pharmacokinetic (PK) BTH is very low with ravulizumab, the risk of pharmacodynamic (PD) BTH and EVH is similar to that of eculizumab [4,6]. The phase 3 trials for the combination of pozelimab (an IgG4 monoclonal antibody) and cemdisiran (a small interfering RNA) are making progress. Even though their method of action is different from C5 blockade and requires a longer induction time, cemdisiran can inhibit C5 for up to 13 months [6]. Furthermore, SB12 and ABP959 are biosimilars of eculizumab that have completed phase 3 studies. Elizaria is a licensed compound exclusively used in Russia [6]. Finally, phase 3 studies show early promise for C3 inhibitors and factor D and factor B inhibitors in PNH patients treated with C5 inhibitors. They are more potent and narrowly selective than pegcetacoplan [6].

## Conclusions

PNH is a rare blood disorder that is caused by a genetic mutation leading to a deficiency of specific proteins on the surface of red blood cells. This deficiency triggers the immune system to attack and destroy these cells, which can result in severe symptoms such as fatigue, organ damage, and even life-threatening complications. The diagnosis of PNH involves flow cytometry and genetic testing, while its treatment options include monoclonal antibodies, blood transfusions, and bone marrow transplants. Several cost-effective and easily accessible treatments are being evaluated to manage and treat PNH. Among these, long-acting C5 inhibitors like ravulizumab and crovalimab show promising results and are more convenient due to their low-volume subcutaneous injections. Furthermore, phase 3 studies have shown early promise for C3 inhibitors and factor D and factor B inhibitors in PNH patients treated with C5 inhibitors, which are more potent and narrowly selective than pegcetacoplan. Effective diagnosis and treatment of PNH require close collaboration between patients and healthcare providers, making it crucial for them to remain informed and up-to-date on the latest advancements in the field.
